# High Fidelity – No Evidence for Extra-Pair Paternity in Siberian Jays (*Perisoreus infaustus*)

**DOI:** 10.1371/journal.pone.0012006

**Published:** 2010-08-09

**Authors:** Phillip Gienapp, Juha Merilä

**Affiliations:** Ecological Genetics Research Unit, Department of Biosciences, University of Helsinki, Helsinki, Finland; University of Jyväskylä, Finland

## Abstract

Extra-pair paternity (EPP) in birds is related to a number of ecological and social factors. For example, it has been found to be positively related with breeding density, negatively with the amount of paternal care and especially high rates have been observed in group-living species. Siberian jays (*Perisoreous infaustus*) breed at low densities and have extended parental care, which leads to the expectation of low rates of EPP. On the other hand, Siberian jays live in groups which can include also unrelated individuals, and provide opportunities for extra-pair matings. To assess the potential occurrence of EPP in Siberian jays, we analysed a large data pool (n = 1029 offspring) covering ca. 30 years of samples from a Finnish Siberian jay population. Paternities were assigned based on up to 21 polymorphic microsatellite markers with the additional information from field observations. We were unable to find any evidence for occurrence of EPP in this species. Our findings are in line with earlier studies and confirm the generally low rates of EPP in related Corvid species. These results suggest that ecological factors may be more important than social factors (group living) in determining costs and benefits of extra-pair paternity.

## Introduction

Extra-pair paternity (EPP) is a widespread phenomenon in birds: it has been found in three quarter of the socially monogamous bird species studied [1]. There is also striking interspecific variation in the rate of EPP, which seems to be related to ecological and social factors, which in turn are also correlated with phylogeny [1], [2], [3]. For example, species or populations with high breeding densities appear to have higher rates of EPP than those with low breeding densities [4], [5], [6], presumably because there are more opportunities for extra-pair matings in high than in low density populations. Consequently, the social system may also influence the rate of EPP, and indeed, the highest rates of EPP are found in group-living and cooperatively breeding Superb Fairy-wrens (*Malurus cyaneus*) where 72% of all offspring are extra-pair offspring and almost all broods contain extra-pair offspring [7], [8]. Another important factor that may affect the rate of EPP is the amount of paternal care provided. The larger the investment of the male is, the greater is the fitness loss faced by unfaithful females if their mates abandon the brood after detecting EPP of their female. Consequently, EPP rates should be lower in species where males provide parental care [9], [10], [11].

Siberian Jays (*Perisoreus infaustus*) are long-lived (on average 4.9 years for breeders and up to 16 years), socially monogamous birds that live in family groups consisting of the breeding pair, so-called retained offspring and additional individuals that are unrelated to the breeding pair [12], [13]. Pair-bonds are stable and divorces occur rarely, if ever [12], [14]. While both retained offspring and unrelated individuals are tolerated within the territory, retained offspring are treated favourably by the parents [e.g. 15], [16].

In this system with stable pair-bonds and ‘prolonged brood care’ [13], [17], females engaging in extra-pair matings would be expected to pay high costs of infidelity since raising a brood on her own or finding a new partner are likely to incur high fitness costs. Also limited possibilities of finding an extra-pair mate can be important as population densities are low (0.1–2 ind/km^2^) and territories are large, 1–4 km^2^ [14], [18], [19]. A female would thus have to travel several kilometers only to sample a reasonable number of potential extra-pair mates. On the other hand, unrelated group members provide candidates for extra-pair matings. Here we analysed a large data set from a long-term study of a Finnish Siberian jay population with over 1000 individuals genotyped at up to 21 microsatellites to test for evidence for extra-pair paternity in this species.

## Methods

### Study species and area

Siberian jays (*P. infaustus*) are comparably small (ca. 80 g) corvids inhabiting boreal forests from Fennoscandia to Siberia. After becoming independent in early summer, juveniles have three options. They either (i) leave their natal family group to join another family group, (ii) establish a territory of their own, or (iii) stay within the natal family group as retained offspring [12], [13]. Groups consist of the breeding pair and up to four additional individuals that are retained offspring or unrelated individuals [13], [18], [20], but neither retained offspring nor do the unrelated group-members help at the nest [13].

**Figure 1:**
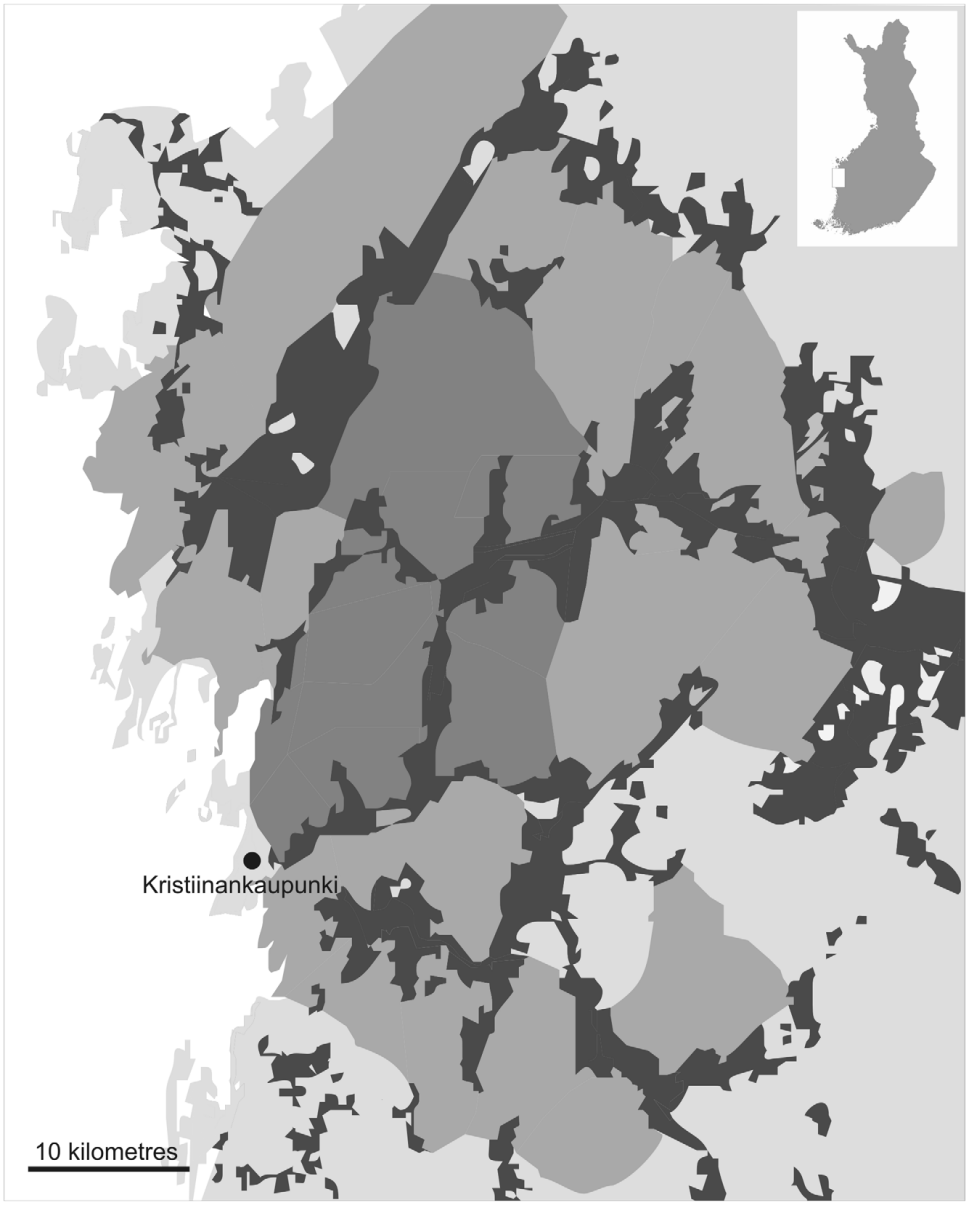
Schematic map of the study area. Dark grey areas indicate open land (mainly arable fields). The study area is indicated by medium gray, with darker medium gray indicating areas where monitoring started in 1992 and earlier. Lighter medium gray indicates areas where monitoring started in 1998 and later. Areas outside the study area are shown in light gray. The inset map shows the location of the study area within Finland.

The study area is located in Ostrobothnia (SW-Finland), near Kristiinankaupunki (62°22′N, 21°30′E). Annual monitoring of the Siberian jay population started in 1974 in a 120 km^2^ sized forest area. Subsequently, the study area has been enlarged and now covers about 1000 km^2^ divided into seven sub-areas with a maximum N-S and E-W distances of about 75 km and 50 km, respectively (Fig. 1). The number of breeding pairs in the area has varied from five in the early years to 50 in later years. For a more detailed description of the study area and fieldwork procedures see [14], [18]. Briefly, during late summer and autumn (July–October) birds are caught at feeding stations, measured, blood sampled (since 1997) and ringed with aluminium and colour rings. In some years additional observations in spring were done. All birds included in this analysis were sexed using molecular markers [21].

Capturing and ringing of birds was approved by the institution that coordinates ringing activity in Finland (Finnish Museum of Natural History), based on the regulation by the Ministry of the Environment (No. 17/5713/2002). Procedures for blood and DNA sampling were approved by the ethical license board of University of Helsinki (No. HY 133-06).

### Paternity analysis and pedigree construction

The original pedigree for this study-population was constructed based on field observations and microsatellite markers [22], [23]. In short, identities of breeding partners were inferred from observations at feeding stations, and the genotypes of all offspring born in a given year were manually matched against all breeding pairs in the same year [18], [23]. Based on the improved genotypic data (see below) and parentage analysis using Cervus 3.0 [24], we re-constructed the pedigree. This was done separately per birth year to avoid the possibility that an individual's offspring could become assigned as its parent. We used Delta-scores, the difference in the logarithms of the likelihood ratios (LOD) of the most likely and the second most likely candidate pair, as a criterion for the confidence of the parentage assignment. Because the number of candidate parents differed between years, critical Delta-scores were calculated on an annual basis using the simulation option in Cervus. We set the proportion of the population sampled to the average annual recapture probability [0.90±0.02, 25]. The genotyping error was assumed to be 1%. This is a conservative estimate since all samples were run twice, read blindly and the resulting genotypes only accepted if the two runs gave identical results. If the results differed the sample was re-ran and re-scored. To test how sensitive our results were to this parameter, we re-run a subsample using genotyping errors of 0.1% and 2%, respectively. No qualitative differences in the assigned parentages were found. All individuals genotyped at less than six (1974–79), less than eight (1980–96) and less than nine loci (1997–2006), respectively, were excluded from the analysis. The threshold values for exclusion differed between periods because the average number of loci typed per individual differed: Initially only individuals after 2005 were genotyped for 21 markers. Not all other individuals could be successfully typed for the new markers due to different sample types (feathers vs. blood). All parents that were (possibly) alive in the given birth year were included as candidate parents in the separate runs. The most likely candidate parents as identified by Cervus were assigned as ‘true’ parents if they were observed as a breeding pair in the individual's birth year. When the most likely candidate parents were not observed as a breeding pair, but the second or third most likely candidate pair was observed as a breeding pair, the latter were assigned as parents. However, in all these cases the Delta-score was small, and no candidate pair could be assigned with 95% confidence. If none of the candidate pairs had a positive LOD-score or were observed together as a breeding pair, no parents were assigned to the individual.

Normally, nests were not located and visited because of the amount of work involved with finding the nests, but also to minimise the disturbance which can increase the risk of nest predation [e.g. 26], [27]. However, in total 37 broods were ringed in the nest. Of these, 40 offspring from 23 broods could be included in the paternity analysis. In three cases no DNA-sample from the social mother was available, but they were included regardless. Paternity analysis was carried out using Cervus assuming the social mother was the ‘true’ mother. Again, paternity assignments were carried out with the known genetic mothers separately per birth year since the number of candidate fathers differed between years. We used the same values for proportion of the population sampled and genotyping error as in the parentage analysis.

Genetic samples were obtained from blood samples and for the early years from the tip of collected tail feathers. DNA was extracted using DNeasy Blood & Tissue kit according to the manufacturers' instructions (Qiagen). The individuals were genotyped for 21 previously reported markers (Table 1). Briefly, the markers were divided into groups (panels) of 3–4 markers based on the size of the PCR products and the labels used. PCR amplification was carried out in similar conditions (2 pmol of each primer, 1× Qiagen multiplex mastermix, 0.5× Q-Solution, and approximately 30 ng of DNA in a total volume of 10µl) and the same cycling profile (15 min at 95°C, followed by 30 cycles 30 s at 94°C, 90 s at 56°C and 60 s at 72°C and a final extension for 10 min) for all markers, using a commercial multiplex PCR kit (Qiagen). The PCR products were diluted 1∶750 with MQ-water and mixed with Et-ROX 400 standard (GE Healthcare, Life Sciences) according to the manufacturer's instructions and resolved in a MegaBace 1000 capillary sequencer (GE Healthcare, Life Sciences). Genotypes were scored with the Fragment Profiler 1.2 program (GE Healthcare, Life Sciences). Not all birds could be scored for all markers due to low quality and low concentration of DNA. This was mainly the case for the old feather samples.

**Table 1:** Microsatellite loci used for pedigree re-construction and extra-pair paternity analyses.

Locus	A	n	H_O_	H_E_	PIC	N-Excl_1_	N-Excl_2_	Ref.
SJ103	18	929	0.872	0.895	0.885	0.3507	0.2121	[21]
SJ104	6	950	0.323	0.322	0.307	0.9458	0.8208	[21]
SJ105	5	909	0.482	0.503	0.408	0.8730	0.7776	[21]
SJ106	8	953	0.787	0.802	0.774	0.5650	0.3876	[21]
SJ107	10	951	0.693	0.698	0.653	0.7084	0.5346	[21]
SJ109	9	926	0.726	0.755	0.715	0.6450	0.4682	[21]
SJ110	4	934	0.668	0.653	0.587	0.7706	0.6176	[21]
SJ111	5	921	0.562	0.587	0.523	0.8218	0.6758	[21]
SJ112	4	895	0.191	0.302	0.257	0.9544	0.8706	[21]
SJ114	5	888	0.498	0.505	0.410	0.8718	0.7754	[21]
SJ116	3	861	0.48	0.482	0.379	0.8841	0.8032	[21]
SJ115	4	872	0.429	0.505	0.432	0.8724	0.7536	[21]
MJG1	2	1034	0.414	0.405	0.323	0.9179	0.8384	[37]
PER1	7	1034	0.538	0.539	0.490	0.8438	0.6916	[37]
Ppi1	4	1008	0.57	0.543	0.485	0.8494	0.7050	[37]
PPi2	5	1029	0.747	0.728	0.681	0.6910	0.5158	[37]
LTML7	3	1007	0.367	0.37	0.302	0.9316	0.8488	[37]
LTML8	13	1022	0.86	0.848	0.831	0.4643	0.3000	[37]
CK1B5D	2	1003	0.5	0.495	0.373	0.8774	0.8137	[37]
CK2A5A	16	1033	0.803	0.807	0.787	0.5295	0.3557	[37]
CKL5	12	1010	0.82	0.815	0.792	0.5296	0.3558	[37]

Number of alleles (A) and number of individuals genotyped (n), observed (H_O_) and expected (H_E_) heterozygosity, non-exclusion probability for first (N-Excl_1_) and second (N-Excl_2_) parent (with first parent assigned) per locus. Ref. = reference for original publication describing the loci.

## Results

A total of 1029 genotyped offspring could be included in the parentage analysis. In 177 cases (17.2%) no candidate parents with positive LOD scores were found and these individuals were hence regarded as immigrants into the study area. In the majority of the remaining cases (71%) the most likely candidate pair was observed as a breeding pair, and hence, assigned as parents. In 118 (11.5%) cases the most likely candidate parents had a positive LOD scores but were not observed as an actual breeding pair, but were breeding in different territories with other partners. These are thus possible cases of extra-pair paternity. However, for 76 of these the LOD and the Delta scores were so low that none of the candidate pairs could be assigned for parentage at the 95% confidence level. In the remaining 36 (3.5%) cases, the candidate pairs could be assigned at the 95% confidence level but there are several reasons why we think that none of them provides good evidence for extra-pair paternity. 1) In eight cases one or both candidate parents could already have died, because they were observed in the years before but not in the year when the individual in question was born. 2) In the majority of cases, the male or female would have had to travel quite far (mean = 14.7 km, range = 1.6–41.3 km) to seek the extra-pair mating, further than the average natal dispersal distance (mean = 5.0 km (males), 5.9 km (females), [28]) and also further than the neighbouring territories. 3) In none of these cases social mother would have been the genetic mother, and the offspring in question were never observed in the assumed natal territory. This means that all extra-pair offspring would have dispersed in their first summer, which seems very unlikely given that a half to two thirds of the fledglings stay with their parents [20]. It becomes even more unlikely when one considers that extra-pair offspring should be of higher quality [29] and that it is the low-quality, sub-dominant offspring which are more likely to disperse during the first summer [20].

Four out of 40 offspring with known social father could have been EPP-offspring. However, in the years when three of these offspring hatched critical LOD and Delta-scores were low (Table 2). This was because not all individuals could successfully be genotyped for 21 markers and the number of candidate fathers was small. This was true even for strict (95%) confidence, which means that almost all candidate fathers were possible ‘true’ fathers. It would hence be difficult to assign any extra-pair father as ‘true’ father with any confidence and consequently there is no good evidence for these three offspring really being EPP-offspring. In 2002 and 2003 critical LOD and Delta scores were reasonably high to distinguish between likely and unlikely candidates (Table 2). 19 nestlings from eight broods in these years could be included in the analysis and in only one case an extra-pair candidate father was the most likely father. However, both this candidate father as well as the social father mismatched at one locus with the offspring and neither of them would have been likely father if tested on their own without knowledge of the social mother's genotype.

**Table 2:** Minimum number of loci for inclusion in analysis, median number of loci typed per individual, number of candidate fathers and critical LOD-scores and Delta-values (95% confidence) of the paternity analysis of offspring with known social parents, i.e. being ringed in the nest, separately per year.

				Father alone		Father given known mother	
Year	minimum	mean	candidate fathers	critical LOD	critical Delta	critical LOD	critical Delta
1976	6	10.4	17	1.15	0.25	−4.50	0.00
1980	6	13.5	15	0.27	0.16	−4.50	0.00
1984	8	12.4	6	−4.00	0.00	−9.00	0.00
1987	8	16.1	12	−1.00	0.00	−6.00	0.00
1990	8	17.3	24	1.81	0.77	−3.00	0.00
1991	8	18.5	29	2.85	1.08	−2.75	0.00
1992	8	19.2	25	2.00	0.84	−3.50	0.00
1993	8	19.1	36	3.53	1.48	−1.88	0.00
1994	8	18.8	41	3.57	1.72	−1.13	0.00
2002	8	20.8	116	5.54	2.57	2.19	1.01
2003	8	20.8	112	5.90	2.41	2.74	0.85

Locating nests did not belong to the standard field work procedure and was done only in some years.

## Discussion

We found no evidence for extra-pair paternity (EPP) in our study population despite the fact that data set covered over 30 years and more than 1000 offspring genotyped for up to 21 microsatellite markers. This concurs with an earlier study of the same population [23] and the results of an analysis of a smaller data set of five large families (349 individuals) genotyped for 117 microsatellite loci from the same population [30] that did not find a single case of EPP. Likewise, in a Swedish study of Siberian jays, no evidence for extra-pair paternity was found [31]. Hence, our results – together with similar evidence from earlier studies – provide little evidence to support the possibility that extra-pair paternity in Siberian jays would be common, or in matter of fact, occurring at all. Of course, we cannot strictly rule out the possibility that single cases of extra-pair paternity may have gone undetected. Yet, even if extra-pair paternity in Siberian jays would occur, its frequency must be very low.

Due to the limited number of loci typed and the small number of candidate fathers included in extra-pair paternity analysis, critical LOD- and Delta-scores – and thereby our ability to detect extra-pair paternity – were low for several years. However, even in years when critical these scores were reasonably high to distinguish in between likely and unlikely candidates, little evidence for extra-pair paternity was found. Another reason why our analyses might underestimate the rate off EPP in Siberian jays is that most of the offspring were sampled after they had left the nest. This means that young that died or dispersed outside the study area before sampling were not available for analyses, and reduces the likelihood of detecting EPP. However, focussing on those 23 broods ringed in the nest and available to analyses, this possible bias could be eliminated: none of those offspring were very likely EPP-young. Hence, if some EPP young were missed due to early mortality or dispersal, their frequency is likely to have been low.

If extra-pair paternity in Siberian jays is so rare as our data and earlier analyses suggest, possible reasons for this become of interest. Phylogeny explains over 50% of the among species variation in extra-pair paternity [1]. Although it is not clear whether this is due to ecological similarity of related species or an actual phylogenetic constraint, it is interesting to note that our result of no extra-pair paternity in Siberian jays is in line with the generally low rates of extra-pair paternity in other corvids [0–1.4% extra–pair offspring, 32,33,34,35,36].

Providing ‘prolonged brood care’ is beneficial in Siberian jays since retained offspring have improved survival prospects [37] and higher life-time reproductive success as compared to dispersing offspring [28], [31]. Successful ‘prolonged brood care’ requires both parents and an unfaithful female being deserted by its male may face brood loss and consequently high fitness costs.

While the generally low breeding densities in Siberian jays should reduce the ‘availability’ of extra-pair mates, this may be counteracted by the presence of unrelated males within the group. However, unrelated individuals within a family group have been forced to leave their natal family by dominant siblings [20]. Since they are therefore likely to be of low ‘quality’ (phenotypic or genetic), they may be unattractive candidates for extra-pair matings. Furthermore, most of the unrelated group-members will leave the group in spring to attempt to establish their own territories.

The observed absence or very low frequency of EPP in Siberian jays could hence be easily explained by the high costs of EPP due to their breeding system and the reduced choice of extra-pair partners.
